# Microscopic measurement of the local deformation field establishes the mechanistic origin of the fatigue threshold for soft brittle materials

**DOI:** 10.1039/d6sm00197a

**Published:** 2026-04-09

**Authors:** Umut Altuntas, Chenzhuo Li, John Martin Kolinski

**Affiliations:** a Institute of Mechanical Engineering, School of Engineering, EPFL 1015 Lausanne Switzerland john.kolinski@epfl.ch; b Institute of Materials, School of Engineering, EPFL 1015 Lausanne Switzerland

## Abstract

Fatigue fracture, whereby a material fails only under repeated loading cycles, depends strongly on load magnitude. In most materials, the applied load must exceed a threshold value – the fatigue threshold – for the crack to advance each cycle. While recent studies clarify the regimes of soft polymer response to cyclic loading, the interplay between crack tip strain fields, irreversible deformation, and energy dissipation remains unclear. Here, we subject polyacrylamide hydrogels and polydimethylsiloxane (PDMS) elastomers to controlled cyclic loading, while directly resolving crack tip strain fields with particle tracking microscopy. An irreversible deformation zone emerges at the crack tip with a load-amplitude dependent structure. Below the fatigue threshold, the zone is compressive, and progressively accumulates without crack advance; this demonstrates that sub-threshold deformation is not fully reversible. Above threshold, a tensile plastic zone grows and co-propagates with the crack tip. Crack tip opening displacement (CTOD) measurements of the energy release rate *G* show that the applied strain energy evolves with cycle count under constant stretch amplitude loading. Below threshold, *G* remains nearly constant; above threshold, expansion of the tensile plastic zone modifies the CTOD geometry, producing a measurable decrease in *G*. Consistent across both material systems in the brittle limit, these results suggest that irreversible deformation—shielding the crack below threshold and governing growth rate above it—may be a general hallmark of soft brittle material fatigue. These findings open pathways toward rational design of fatigue-resistant hydrogels and elastomers for soft robotics, biomedical devices, and stretchable electronics.

## Abstract 2

Direct crack tip strain imaging reveals irreversible plastic zones governing soft material fatigue, both below and above the fatigue threshold.

## Graphical abstract

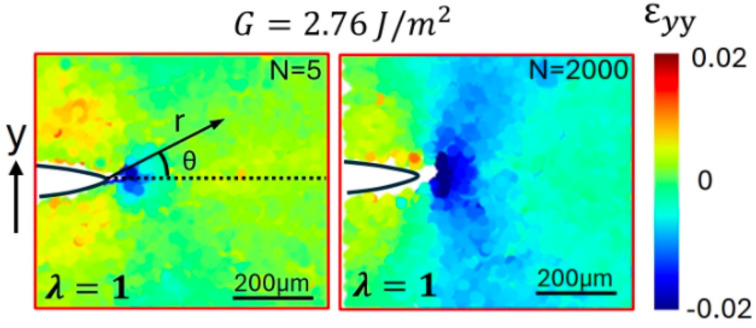

## 1. Introduction

Why does a crack propagate under cyclic loading at an energy release rate below the fracture toughness of the material? The fact that cracks respond differently to the same magnitude of applied load when applied cyclically or monotonically represents a major deviation from Griffith's classical theory. While fatigue fracture is well documented in metals, composites, and ceramics,^1^ the precise mechanisms driving crack growth in amorphous soft materials remain obscured. Consequently, most current fatigue models are empirical, relying largely on curve fitting rather than capturing the underlying physical processes.^2–4^ Fatigue life is typically characterized by measuring the stress amplitude as a function of mean number of cycles before failure (*S*–*N* curves),^5^ and stress-stretch curves to quantify mechanical response.^6–8^ While these methods allow for quantitative lifespan assessment and reveal global load–unload hysteresis, they characterize the bulk response, effectively treating the crack tip as a “black box” and obscuring the local physics driving failure.

A lack of micro mechanistic insight is particularly pronounced in soft materials. Unlike metals and ceramics, where damage mechanisms such as dislocation motion or microvoid coalescence are well understood,^1^ soft materials like hydrogels and elastomers fail through distinct stress deconcentration processes, including polymer chain scission, viscoelastic energy dissipation, and network rearrangement.^9,10^

As these materials are increasingly integrated into soft robotics,^11,12^ biomedical implants,^13^ and flexible electronics,^14,15^ understanding their fundamental fatigue limits is essential. Recent research has focused primarily on material formulation strategies to enhance resistance, such as developing fatigue free ionic skins,^16^ engineering loose crosslinking networks,^17^ controlling crystallinity,^18^ or reinforcing elastomers through geometrical modifications.^19–21^ These improvements are predominantly validated using pure shear tests^22–27^ to demonstrate increases in the macroscopic fatigue threshold. Yet, these global energy measurements often yield inconsistent values depending on the methodology^28–30^ and, crucially, fail to resolve how the crack propagates or stabilizes at the microscale. As a result, the specific link between irreversible deformation and crack growth, and how the local energy release rate (*G*) evolves during cyclic loading, remains unknown.

In this work, we use polyacrylamide (PAA) hydrogels and PDMS as canonical model systems for soft brittle fracture, playing a role analogous to that of copper for metals or silica for ceramics. The optical transparency and thoroughly characterized mechanical response of these polymeric materials enable high resolution imaging of crack tip phenomena that remain obscured in conventional opaque materials.^31,32^ We subject pre-cracked specimens to displacement-controlled cyclic loading while concurrently imaging the near tip deformation fields using particle tracking.^33^ At the microscale, we observe nonlinear crack propagation and identify a distinct threshold value for fatigue crack propagation. We demonstrate that sub-threshold loading generates a compressive plastic zone, thereby inhibiting propagation, whereas above threshold loading produces a tensile plastic zone that evolves ahead of the crack tip as cycle count increases. Furthermore, by measuring the energy release rate (*G*) *via* the crack tip opening displacement (CTOD), we reveal that the local driving force decreases during cycling and approaches a steady state. This evolution reflects a critical redistribution of energy between surface creation and plastic deformation. The observation of similar deformation and propagation behaviors across two distinct material chemistries suggests a unified mechanistic framework governing the onset of fatigue fracture in soft brittle polymers.

## 2. Experimental methods

### 2.1. Material preparation

Hydrogels embedded with tracer particles (0.005 wt%) were prepared with a final composition of 13.8 wt% acrylamide monomer and 2.7 wt% bisacrylamide crosslinker.^34^ Free-radical polymerization was initiated by adding 0.2 wt% ammonium persulfate (APS) and 0.02 wt% tetramethylethylenediamine (TEMED), with all concentrations defined with respect to the final hydrogel solution. The solution was cured in molds for 2 hours at 23 °C to form sheets of 200 µm thickness. To enable digital tracking of local deformation fields, rigid tracer particles (1 µm diameter polystyrene colloids, Sigma Aldrich) were dispersed within the matrix prior to polymerization.^35^

PDMS samples were prepared by mixing a silicone elastomer base and curing agent (Sylgard 184, Dow Corning) at a 10 : 1 weight ratio. The same 1 µm diameter polystyrene colloids were added to the crosslinker component and thoroughly mixed before the two components of the Sylgard were ultimately mixed together. The components were stirred for at least 5 minutes to ensure homogeneity and degassed under vacuum for 30 minutes to eliminate trapped air bubbles. The mixture was cast between two glass plates separated by 200 µm spacers and cured at 65 °C for 2 hours. After curing, specimens were cut into 30 × 10 mm rectangular strips.

### 2.2. Fatigue testing and optical imaging

**Fig. 1:**
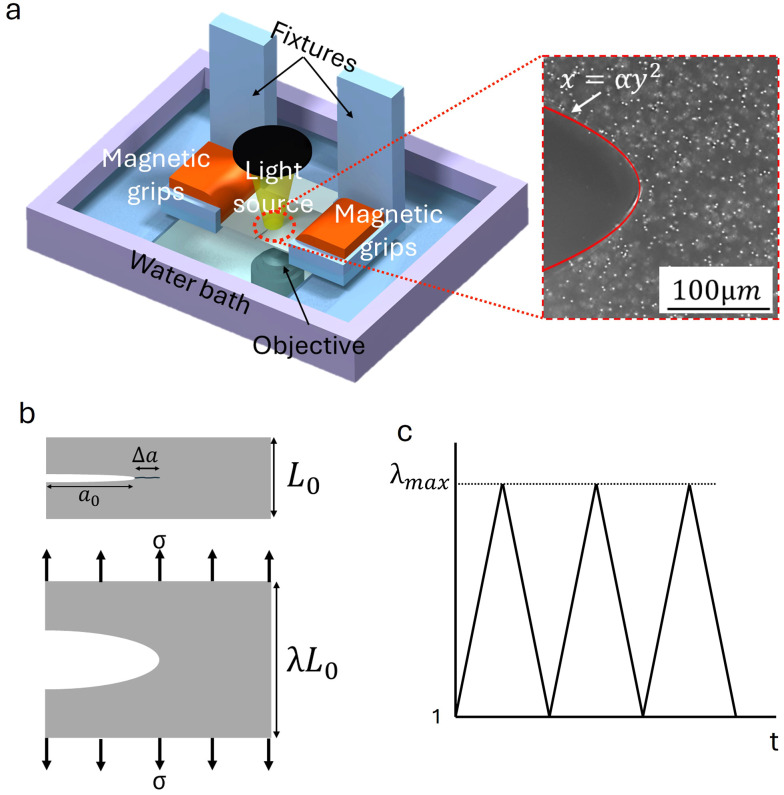
(a) Schematic of the experimental setup. A hydrogel sample with a thickness of 200 µm, embedded with 1 µm rigid particles, is gripped by magnetic fixtures in a water bath to prevent dehydration and enhance image quality using a water immersion objective. A captured 2D image of the stretched hydrogel or PDMS shows a parabolic crack opening. The crack tip opening, highlighted in red, follows the function *x* = *αy*^2^, where *α* is used to calculate the energy release rate. (b) Schematic of the sample in unstretched and stretched configurations. The diagram indicates the initial crack length *a* and crack growth Δ*a* under cyclic loading. (c) Loading profile of hydrogel and PDMS during fatigue experiments. The test begins at minimal prestress, ensuring sample flattening and suppressing out of plane motion during minimum stretch phases.

Fatigue experiments were conducted on pre-cracked samples using the loading apparatus shown schematically in Fig. 1a. Samples were prepared with an initial gauge length *L*_0_, and initial crack length *a*_0_, as shown in Fig. 1b.

To prevent dehydration of the hydrogels and to match the refractive index for imaging, all tests were performed in a water bath for both hydrogel and PDMS, as illustrated in the schematic.

Magnetic grips with high friction silicone pads were used to ensure constant grip force, and strictly prevent slippage during cyclic loading. Cyclic loading was applied to the sample using a triangular waveform to drive a servo motor, cycling between a minimum stretch *λ*_min_ ≈ 1 and a maximum stretch *λ*_max_. Crucially, a minimal prestress was maintained at the bottom of each cycle to keep the sample taut and suppress out of plane buckling, which is common in thin soft membranes (Fig. 1c). High resolution images of the crack tip region were acquired using a water immersion objective and a blue LED light source to minimize optical distortions.

### 2.3. Crack tip analysis

The applied energy release rate (*G*) was determined locally using the crack tip opening displacement (CTOD) method. The crack tip in these soft brittle solids forms a parabolic profile described by *x* = *αy*^2^, where *y* is the half opening height and *x* is the distance behind the crack tip; a typical image with the CTOD illustrated in the red curve is shown in Fig. 1a at right. The measured CTOD is then used to extract the curvature parameter *α via* a parabolic fit to the profile data. From this fit, *G* is calculated as:1
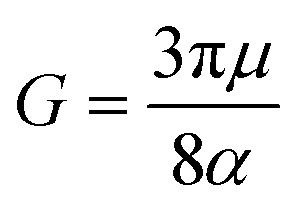
where *µ* is the shear modulus of the material.

## 3. Results

**Fig. 2:**
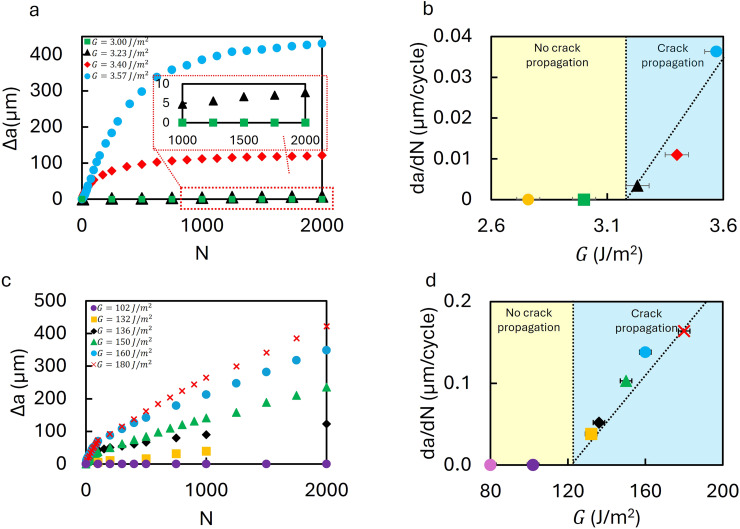
(a) Crack extension Δ*a* (µm) as a function of cycle number *N* for hydrogels at *G* = 3.00, 3.23, 3.40, and 3.57 J m^−2^ (CTOD derived at *N* = 1 unless noted). Three behaviors are observed: no measurable crack growth at *G* = 3.00 J m^−2^; slow, nearly linear growth just above threshold at *G* = 3.23 J m^−2^; and rapid initial growth followed by deceleration and convergence to a steady state regime at *G* = 3.40 and 3.57 J m^−2^. (b) Steady state fatigue crack growth rates (d*a*/d*N*) for hydrogels as a function of *G* for the conditions in (a). Rates are obtained from linear fits to the steady state region of the Δ*a*–*N* curves (see SI Fig. S1). The vertical line marks the estimated fatigue threshold. (c) Crack extension Δ*a* (µm) for PDMS at *G* = 102, 132, 136, 150, 160, and 180 J m^−2^. Analogous to hydrogels: no growth at *G* = 102 J m^−2^, nearly linear growth just above threshold at *G* = 132 J m^−2^, and rapid–then–decelerating growth converging to steady state at higher *G*. (d) Steady state d*a*/d*N* for PDMS *vs. G*; the vertical line indicates the PDMS fatigue threshold. Cycle by cycle evolution and convergence of *G* during fatigue are shown in SI Fig. S2.

To understand the dynamic response of the materials under cyclic loading, we first examined the fatigue crack propagation behavior of hydrogels subjected to energy release rates of 3.0, 3.23, 3.44, and 3.57 J m^−2^, as shown in Fig. 2a. Unless otherwise noted, the energy release rate *G* quoted for each experiment corresponds to the CTOD derived value at the first maximum stretch frame (*N* = 1). The cycle by cycle evolution of *G* is shown in SI Fig. S2, demonstrating its decrease with cycling and convergence to a steady state value.

At 2.76 and 3.0 J m^−2^, no crack propagation is observed after several thousand cycles, indicating the existence of a fatigue threshold in brittle hydrogels. When the energy release rate increases to 3.23 J m^−2^, crack propagation becomes evident. The growth appears nearly linear from the onset, possibly because the applied strain energy is just above the threshold, sufficient to initiate slow crack growth but insufficient to drive a clearly distinguishable nonlinear regime. In addition, the nonlinear phase associated with the decrease in applied energy may occur over a very short timescale or within a few cycles, making it difficult to capture even with high resolution microscopy.

When the applied energy exceeds 3.23 J m^−2^, the nonlinear characteristics of fatigue crack propagation become more pronounced. This nonlinear behavior intensifies with increasing applied strain energy. Initially, crack growth is rapid and nearly linear, but the rate gradually decreases over successive cycles *N* until it eventually stabilizes, reaching a steady state regime around the 1000th cycle. This response coincides with the decrease and convergence of *G* shown in SI Fig. S2. The steady state crack propagation rates corresponding to each energy level are summarized in the graph in Fig. 2b. The detailed procedure used to identify the steady-state regime, and to calculate the corresponding propagation rates, is depicted in SI Fig. S1. In this regime, Δ*a*–*N* curves fitted to a linear function to obtain the reported steady-state crack growth rate. A similar trend is observed in PDMS, as shown in Fig. 2c. Effective energy release rates of 80, 102, 132, 136, 150, 160, and 180 J m^−2^ were applied to the samples. At 80 and 102 J m^−2^, no crack propagation is observed, indicating the existence of a fatigue threshold for brittle elastomers, as well. Once the applied strain energy exceeds this threshold (approximately 120 J m^−2^), crack propagation begins. The overall behavior mirrors that of hydrogels: an initial rapid, nearly linear growth phase is followed by a decline in propagation rate and transition to a steady state regime. However, in PDMS, this transition is more abrupt than in hydrogels. For applied strain energies just above the threshold, the CTOD derived *G* remains nearly constant during cycling, whereas for larger applied energies, *G* decreases as the tensile plastic zone develops (see SI Fig. S2).

Once the transition to a steady-state crack growth regime occurs, the crack growth rates for the various PDMS experiments can be quantified using the same procedure as that applied to the hydrogel crack advance curves; these measurements are shown in Fig. 2d. Once the threshold is exceeded, crack growth initiates abruptly and increases with the applied energy release rate. The observed decrease in *G* during cycling is directly related to the growth of plastic deformation zones in front of the crack tip; the plastic deformation zones alter the CTOD geometry and reduce the effective applied energy. When the applied strain energy is relatively small, this effect is limited, and *G* remains constant or decreases only slightly; at higher energies, the effect is pronounced. SI Fig. S6 and S7 provide detailed strain field maps during the entire experiments, linking the observed plastic zones to the evolution of *G*.

**Fig. 3:**
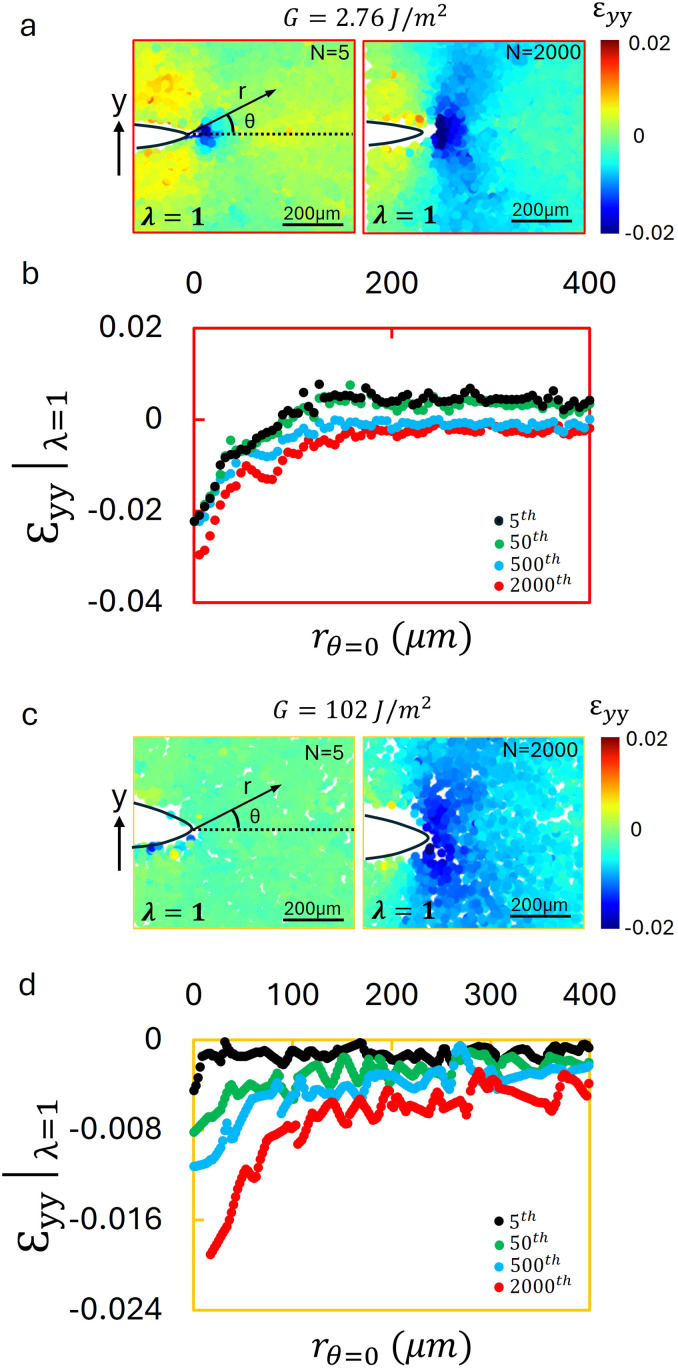
Compressive strain fields in hydrogel and PDMS at the minimum stretch (unloaded) state under subthreshold loading without crack propagation. (a) The *ε*_*yy*_ field, measured by 3D particle tracking,^33^ at the *N* = 5 and *N* = 2000 cycles for hydrogel at an effective energy release rate *G* = 2.76 J m^−2^. A compressive zone is already visible at *N* = 5, and grows larger by *N* = 2000. (b) The *ε*_*yy*_ strain trace along the dashed line in (a) for the 5th, 50th, 500th, and 2000th cycles, showing that the compressive field intensifies, and extends deeper into the material with continued cycling. (c) The *ε*_*yy*_ field for PDMS at an effective energy release rate *G* = 102 J m^−2^ at the 5th and 2000th cycles. A compressive zone develops ahead of the crack tip in the absence of crack growth, analogous to the observed response of the hydrogel material. (d) Corresponding *ε*_*yy*_ distributions along the dashed line in (c) for the 5th, 50th, 500th, and 2000th cycles, indicating accumulation of compressive strain with cycling. Additional *ε*_*yy*_ fields at different applied strain energies and swelling control analyses are provided in SI Fig. S3–S5.

To investigate the microstructural changes driving the observed sub-threshold behavior, the *yy*-component of the strain field, *ε*_*yy*_, is calculated by particle tracking^33^ at the minimum stretch ratio. *ε*_*yy*_ field graphs for the effective energy release rate (*G* = 3 J m^−2^) are analyzed for cycles *N* = 5 and *N* = 2000, and graphed in Fig. 3a. A compressive strain zone is visible ahead of the crack tip at *N* = 5; its size increases substantially by *N* = 2000.

The corresponding strain distributions along *θ* = 0 confirm that the compressive field intensifies and penetrates deeper into the material with continued cycling, as plotted in Fig. 3b. This persistent compressive zone indicates the accumulation of plastic deformation even though the applied energy remains below the fatigue threshold. Additional strain fields at different subthreshold energies confirm that the size and magnitude of the compressive zone depend on the effective applied *G*, as shown in SI Fig. S3. Furthermore, swelling or deswelling in the water bath does not account for this effect, as shown in SI Fig. S4.

A similar trend is observed in PDMS. The *ε*_*yy*_ field at *G* = 102 J m^−2^ for *N* = 5 and *N* = 2000 shows the development of a compressive zone, as presented in Fig. 3c. While negligible at *N* = 5, a distinct compressive zone develops by *N* = 2000, although its growth is slower than in hydrogels. The corresponding strain profiles in Fig. 3d show a progressive shift toward compression, with the maximum compressive strain increasing over successive cycles. These results indicate that PDMS undergoes irreversible deformation when subject to sub-threshold fatigue loading without crack growth, as was observed for the hydrogel sample. Swelling or deswelling does not contribute to this behavior, as shown in SI Fig. S5.

Importantly, the compressive fields emerge in the unloaded (minimum stretch) state, implying that they represent residual plastic deformation rather than transient elastic recovery. This phenomenon is reminiscent of fatigue crack closure in metals, where contact between crack faces during unloading reduces the effective stress intensity range and delays crack growth.^36–39^ Although full crack face contact is unlikely in soft materials due to their low modulus, we posit that the observed compressive zones represent a soft material analog of partial crack closure.

**Fig. 4:**
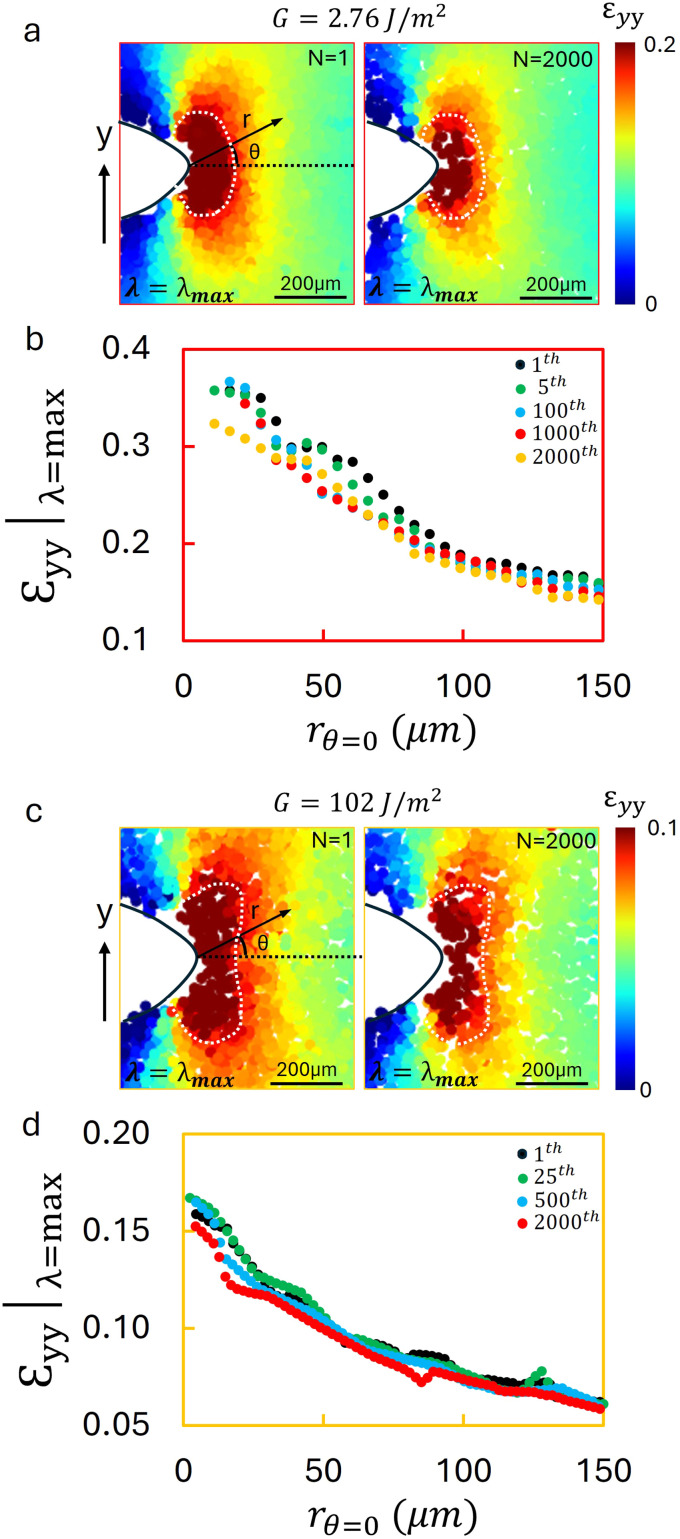
Strain fields and distributions for hydrogel and PDMS at maximum stretch under cyclic loading without crack propagation. (a) The *ε*_*yy*_ field for hydrogel at *G* = 2.76 J m^−2^, measured at *N* = 1 and *N* = 2000. A pronounced strain concentration is observed near the crack tip at *N* = 1 (outlined by the white dashed contour, *ε*_*yy*_ > 0.2). By *N* = 2000, the high strain region within this contour has diminished markedly. (b) The strain fields along the dashed line in (a) for cycles *N* = 1, 5, 100, 1000, and 2000, showing a progressive reduction in peak strain and a reduction of the elevated strain zone despite identical applied stretch. (c) The *ε*_*yy*_ for PDMS at *G* = 102 J m^−2^, measured at *N* = 1 and *N* = 2000. A high-strain zone (*ε*_*yy*_ > 0.1, outlined by the dashed contour) contracts as *N* increases in the absence of crack growth. (d) Strain distributions along the dashed line in (c) for *N* = 1, 25, 500, and 2000, indicating that far field strain remains largely unchanged, whereas the near tip strain progressively decreases with cycling. These results demonstrate that even at the same maximum stretch and without crack propagation, the near tip strain field reduces and localizes due to irreversible (plastic) damage accumulation.

To better understand damage accumulation around the crack tip in the absence of crack propagation, we analyzed the *ε*_*yy*_|_*λ *=* *max_, the *yy*-component of the strain at the maximum applied stretch ratio. The *yy*-component of the strain in the hydrogel during the first loading cycle (*N* = 1) under an applied energy release rate of *G* = 2.76 J m^−2^ reveals a pronounced plastic strain near the crack tip, as shown in the first image of Fig. 4a. A white dashed contour outlines the region where the strain exceeds 0.2. After *N* = 2000 cycles, the same contour is depicted atop the *ε*_*yy*_ strain field for direct comparison in Fig. 4a. It is evident that the high strain region diminishes over time.

To quantify this change, the strain distribution along *θ* = 0 (the direction of crack advance) was analyzed as a function of distance from the crack tip, as graphed in Fig. 4b. The peak strain decreases with increasing cycle count, as reflected by the reduced region where the strain magnitude exceeds 0.2. If the deformation were purely elastic, and the applied strain energy were constant, the strain field would be expected to remain unchanged; however, the observed reduction in both the peak strain and the extent of the high strain region, indicates the accumulation of irreversible (plastic) damage around the crack tip, even in the absence of observable crack growth.

A similar trend is observed in PDMS. The strain field during the first loading cycle (*N* = 1) under an effective applied energy release rate of *G* = 102 J m^−2^ displays a comparable pronounced strain concentration at the crack tip, as presented in Fig. 4c. A white dashed contour encloses the region where the strain exceeds 0.1. The image at cycle *N* = 2000 (Fig. 4c, right) shows a clear reduction in the high strain area, with the same contour overlaid for direct comparison. The corresponding strain profiles along the *θ* = 0 direction underscore the tendency of the strain near the crack tip to decrease with repeated loading, even in the absence of crack growth, as shown in Fig. 4d. These observations are consistent with previous birefringence imaging results reported by Li *et al.*,^40^ which also revealed evolving mesoscale strain fields under cyclic loading. Our findings demonstrate that cyclic loading below the fatigue threshold leads to progressive, irreversible damage accumulation near the crack tip in both hydrogels and PDMS, even when no visible crack propagation occurs.

**Fig. 5:**
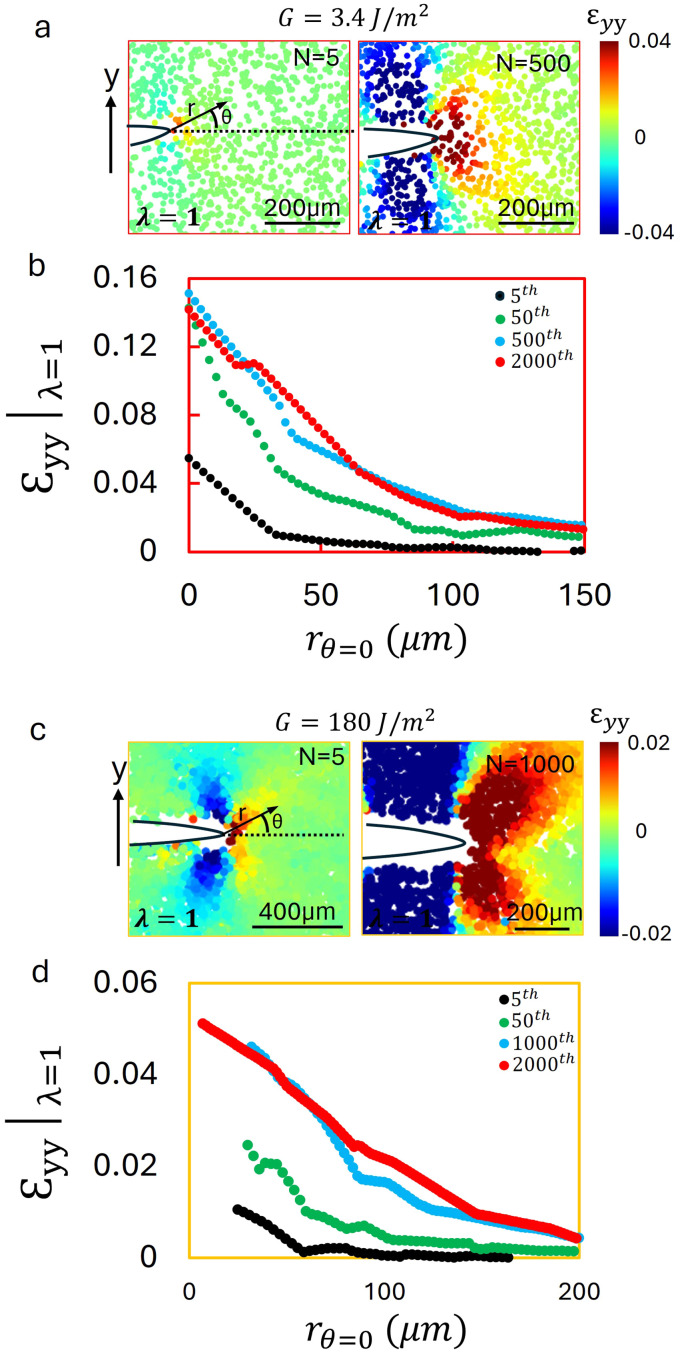
Strain fields and distributions for hydrogel and PDMS at the minimum stretch state during cyclic crack propagation. (a) The *ε*_*yy*_ field measured by 2D particle tracking at *N* = 5 and *N* = 500 for hydrogel at applied strain energy release rates of *G* = 3.4 J m^−2^. A tensile plastic zone evolves ahead of the crack, increasing in both size and magnitude with cycling. A compressive wake also forms behind the crack tip, which likely contributes to the reduction of CTOD by modifying the crack tip geometry. (b) Corresponding strain distributions along the dashed line in (a) for *N* = 5, 50, 500, and 1750 cycles. The tensile zone grows in magnitude and extent but eventually stabilizes, moving forward with the propagating crack. (c) Strain field for PDMS at *G* = 180 J m^−2^ measured by 3D particle tracking at *N* = 5 and *N* = 1000. A tensile plastic zone forms ahead of the crack, accompanied by a compressive wake behind it, analogous to the observed response of the hydrogels. (d) Strain distributions along the dashed line in (c) for *N* = 5, 50, 1000, and 2000 cycles, showing the evolution and stabilization of the tensile zone. These results demonstrate that both hydrogels and PDMS develop tensile plastic zones that co propagate with the crack tip under cyclic loading. The accompanying compressive wakes may further reduce CTOD and contribute to the decrease of *G* observed in SI Fig. S2. Additional strain field maps at different energies are presented in SI Fig. S6 and S7.

To complete the characterization of the near-tip deformation near the fatigue threshold, we examined the *ε*_*yy*_ field for propagating fatigue cracks. For the hydrogel, the *ε*_*yy*_ field at the minimum applied stretch ratio was analyzed for loading cycles *N* = 5 and *N* = 500 at an applied energy release rate of *G* = 3.4 J m^−2^, as shown in Fig. 5a. A small tensile strain zone is observed ahead of the crack at *N* = 5, and its size increases as *N* increases. To quantify this behavior, the strain distribution along *θ* = 0 is graphed as a function of distance from the crack tip in Fig. 5b. As the number of cycles increases, the tensile plastic zone evolves in both magnitude and spatial extent. Eventually, the strain profile converges in shape and amplitude; a similar zone of tensile plastic strain co-propagates with the advancing crack tip. This persistent zone is indicative of network level damage. In addition to the tensile plastic zone ahead of the crack, a compressive wake develops behind the propagating crack tip. This wake modifies the crack tip geometry, and likely contributing to the reduction of CTOD, and thus the decrease in *G* observed during fatigue loading (SI Fig. S2).

A more detailed comparison of the tensile plastic strain field evolution in the hydrogel at applied energy release rates *G* = 3.23 and 3.4 J m^−2^ is provided in SI Fig. S6. These measurements confirm that the extent of the tensile plastic zone correlates with the observed decrease in CTOD. A similar trend is observed in PDMS. The *ε*_*yy*_|_*λ*=min_ field is graphed in Fig. 5c for *N* = 5 and *N* = 1000, with an applied energy release rate of *G* = 180 J m^−2^. A small tensile plastic zone appears ahead of the crack tip by the fifth cycle, and it grows significantly by the 2000th cycle. This behavior closely resembles that of the hydrogel. As in the hydrogel case, we attribute this tensile zone to damage accumulation in the polymer network. A compressive wake is also observed behind the crack, suggesting that the dual characteristics of a leading tensile plastic zone and trailing compressive deformation zone influence the CTOD, and thus *G*, for both of the tested materials. The corresponding strain profiles along *θ* = 0 are shown in Fig. 5d. The evolution of the tensile field reaches a steady-state in both shape and magnitude, and the damage zone co-propagates with the advancing crack tip. SI Fig. S7 presents strain field evolution in PDMS at *G* = 132, 150, and 180 J m^−2^, highlighting the growth of the tensile plastic zones at higher applied strain energies, and the more pronounced decrease of the CTOD.

**Fig. 6:**
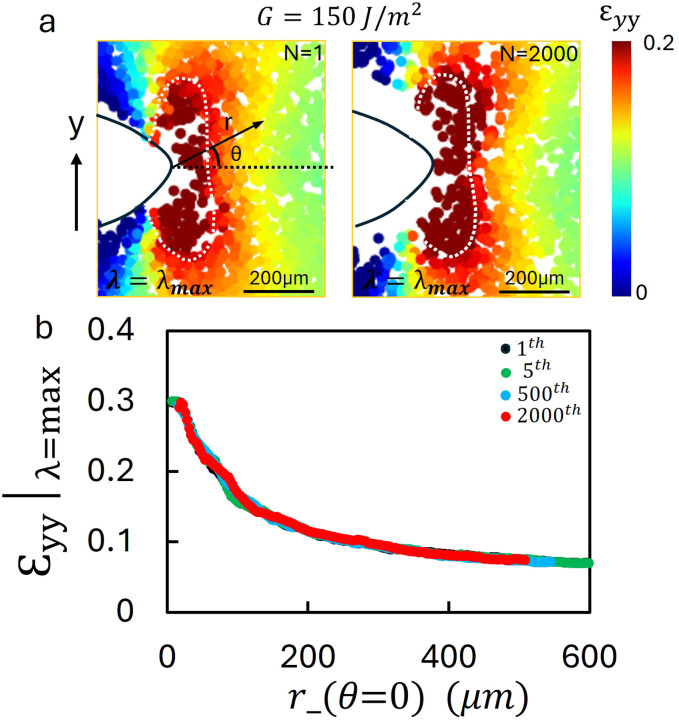
Strain field evaluation and distribution for PDMS under cyclic loading with crack propagation. (a) The *ε*_*yy*_ field at the *N* = 1 and *N* = 2000 cycles for PDMS under an applied energy release rate of 150 J m^−2^ in the stretched configuration. A pronounced strain concentration is observed near the crack tip. The white dashed contour encloses the region with *ε*_*yy*_ > 0.2 from the initial cycle and is overlaid on the 2000th cycle for direct comparison. The high strain region remains aligned with the contour, translating forward with the crack tip rather than shrinking. (b) Strain distribution along the black dashed line in (a) for the *N* = 1, 5, 500, and 2000 cycles. The overall strain distribution and maximum strain values remain largely unchanged during crack propagation. These results indicate that in PDMS the strain field stabilizes and co-propagates with the crack tip even as CTOD decreases, suggesting that the observed reduction of *G* arises from crack tip geometry changes and the influence of the compressive plastic wake rather than from a diminishing strain zone.

To better understand damage accumulation around the crack tip during fatigue crack propagation, the *ε*_*yy*_ field was analyzed at the maximum stretch ratio. During the first loading cycle (*N* = 1) at an applied energy release rate of *G* = 150 J m^−2^, strain is highly concentrated near the crack tip, as shown in Fig. 6a. A white dashed contour circumscribes the region where the strain exceeds 0.2. At the 2000th cycle, the overlaid contour on the *ε*_*yy*_ field highlights the alignment of the elevated strain region, as can be seen in the second image of Fig. 6a. In contrast to the sub-threshold cases, the strain concentration reaches a steady-state that propagates with the advancing crack. To quantify this behavior, the strain distribution along *θ* = 0 is plotted as a function of distance from the crack tip in Fig. 6b. Both the peak strain and the overall distribution remain nearly unchanged as *N* increases, further confirming the co-propagation of a steady-state plastic damage zone. Despite the steady-state nature of the strain field, CTOD measurements decrease as *N* increases. This indicates that the reduction of *G* is not caused by the collapse of the high strain zone, but rather by geometric effects at the crack tip. In particular, the development of a compressive plastic wake behind the crack tip might modify the CTOD geometry, and contribute to the observed decrease in *G* (see SI Fig. S2).

These observations, considering both stretched and unstretched cases, highlight the essential role of irreversible deformation ahead of the crack tip near the fatigue threshold. Once the strain field converges to a characteristic shape, it translates with the crack tip under continued cyclic loading. This steady state damage field appears to be governed by the applied strain energy and plays a critical role in controlling fatigue crack growth in soft brittle materials.

## 4. Discussion

This study reveals that fatigue crack propagation in soft brittle materials, specifically hydrogels and PDMS, follows a nonlinear evolution that depends critically on whether the applied energy release rate is above or below the fatigue threshold. Below threshold, both the hydrogel and the elastomer exhibit plastic deformation without measurable crack growth. A compressive strain zone develops near the crack tip in the unloaded state, expanding over time and suggesting the presence of residual plasticity. Above threshold, this compressive behavior is replaced by a tensile strain field that evolves, stabilizes, and co-propagates with the crack tip. This steady-state field reflects irreversible network damage, with chain scission or localized yielding being candidate material damage mechanisms. Importantly, hydrogels (*G*_th_ ≈ 3 J m^−2^) and PDMS (*G*_th_ ≈ 120 J m^−2^) display analogous responses, despite differences in chemistry and stiffness, suggesting that the observed material responses may be universal in brittle polymeric solids.

In addition to identifying distinct compressive and tensile plastic zones, our experiments provide direct observation of how the effective energy release rate *G* evolves during cyclic fatigue. Conventionally, *G* is assumed to remain constant once applied; however, our CTOD based measurements reveal that *G* decreases as *N* increases, until it ultimately approaches a steady value. The magnitude of this decrease depends on the size of the plastic zone: when plastic deformation is negligible or limited, as near the fatigue threshold, *G* remains nearly constant. By contrast, when large tensile plastic zones develop above threshold, the altered CTOD geometry leads to a measurable reduction in *G*. We propose that both the forward tensile plastic zone and the compressive wake behind the propagating crack tip contribute to this effect, together reducing CTOD and redistributing applied energy between surface creation and plastic deformation. These observations highlight the importance of crack-tip deformation in controlling fatigue crack growth (SI Fig. S2, S6 and S7). This observation is consistent with the concept that fatigue crack propagation is governed by the energy stored within the crack-tip process zone rather than solely by the applied macroscopic energy release rate.^41^ (SI Fig. S2, S6 and S7). Interestingly, in PDMS the near tip strain field at maximum stretch remains essentially unchanged once a steady state damage zone has formed (Fig. 6), even though CTOD decreases. This indicates that the reduction of *G* is not due to the collapse of the strain field itself but rather to geometric effects associated with crack tip opening and the compressive plastic wake.

Our results challenge the conventional assumption that sub threshold loading in soft materials is purely elastic and fully reversible. Instead, we show that irreversible deformation and damage can accumulate even in the absence of crack growth, a phenomenon reminiscent of crack closure and shielding effects observed in metals.^36,37,39^ In both material systems studied, the strain fields ultimately converge to a characteristic spatial profile once crack propagation begins, and this profile translates with the crack tip. This suggests that fatigue crack growth in soft materials is governed not only by the applied energy release rate, but also by the ability of the material to form a steady-state damage zone. The cycle by cycle decrease of *G* (SI Fig. S2) and the evolution of strain fields across different energies (SI Fig. S3–S7) together establish that these effects are robust and reproducible.

Our findings point toward a potential strategy for improving fatigue resistance in soft materials: if a material or structure can be designed to actively redistribute or reduce strain concentration at the crack tip during early stages of cyclic loading, fatigue damage could be delayed or suppressed. For example, engineered toughening mechanisms such as sacrificial bonds, crack tip geometry, or viscoelastic dissipative elements could be tuned to trigger only once crack propagation initiates, thereby reducing strain gradients and local energy dissipation. This approach mirrors strategies used in hard materials (*e.g.*, transformation toughening or crack deflection), but requires soft material specific implementations that preserve deformability while controlling crack tip mechanics.

Interestingly, a similar concept has been demonstrated in collagen based biological tissues, where Tang *et al.*^42^ showed that chemically fixed collagenous matrices can resist fatigue crack growth even in the presence of pre existing flaws. This flaw insensitive behavior arises from a dense and disordered collagen fiber network that enables dynamic strain redistribution and suppresses damage localization. These parallels suggest that strategies inspired by biological architectures may offer valuable design guidelines for fatigue resistant soft polymers.

More broadly, our results may motivate the development of predictive models of fatigue crack growth in soft polymers that incorporate evolving plastic fields, instead of relying solely on global stretch or energy thresholds. Future studies could explore how chemistry, crosslink density, or network architecture influence the shape, size, and dynamics of the plastic zone, as well as its role in crack shielding and propagation rate. By identifying and exploiting mechanisms that manipulate crack tip strain distribution,^10,43–45^ the design of next generation fatigue resistant soft materials becomes a tractable and mechanistically guided goal.

## Conflicts of interest

The authors declare no conflicts of interest.

## Supplementary Material

SM-022-D6SM00197A-s001

## Data Availability

Data will be made available upon request. Supplementary information (SI) is available. See DOI: https://doi.org/10.1039/d6sm00197a.
