# Supplementary material for: Microscopic measurement of the local deformation field establishes the mechanistic origin of the fatigue threshold for soft brittle materials
Source: Soft Matter. 2026 Apr 9;22(17):3165–73. doi: 10.1039/d6sm00197a (PMC13085860; doi:10.1039/d6sm00197a)
Supplement: SM-022-D6SM00197A-s001 [file SM-022-D6SM00197A-s001.pdf]

This journal is © The Royal Society of Chemistry 2026

Supplementary information for "Microscopic measurement of the local deformation field establishes the mechanistic origin of the fatigue threshold for soft brittle materials" by Altuntas et. al.

Supplementary Figure S1 – Crack propagation in hydrogel at 3.57 J/m^2

To understand the macroscopic fatigue behavior, the evolution of crack extension (Δa) as a function of the number of cycles was tracked for a hydrogel sample subjected to an applied energy release rate of 3.57 J/m^2, as shown in Figure 1. The curve reveals three characteristic regimes of fatigue crack propagation. In the initiation regime, the crack growth rate is nearly constant and relatively fast. At this stage, plastic deformation around the crack tip is minimal, and almost all of the applied energy is consumed as free surface energy driving crack extension. As cycling progresses, a transition regime emerges. Plastic deformation begins to develop near the crack tip, and the applied energy is now distributed between free surface creation and the initiation, growth, and evolution of the plastic zone. This redistribution reduces the effective energy available for crack advance, leading to a decrease in the crack growth rate. Finally, a steady-state regime is reached, where a balance forms between free surface energy and plastic zone energy. In this regime, crack propagation stabilizes and the growth rate becomes constant. The steady-state crack growth rates reported in the main text (Figure 2b,d) are derived from this regime by applying linear interpolation to the Δa–N data. Most previous studies, limited by mm–cm scale spatial resolution, missed the initiation and transition regimes and reported only steady-state crack propagation for a given applied energy.

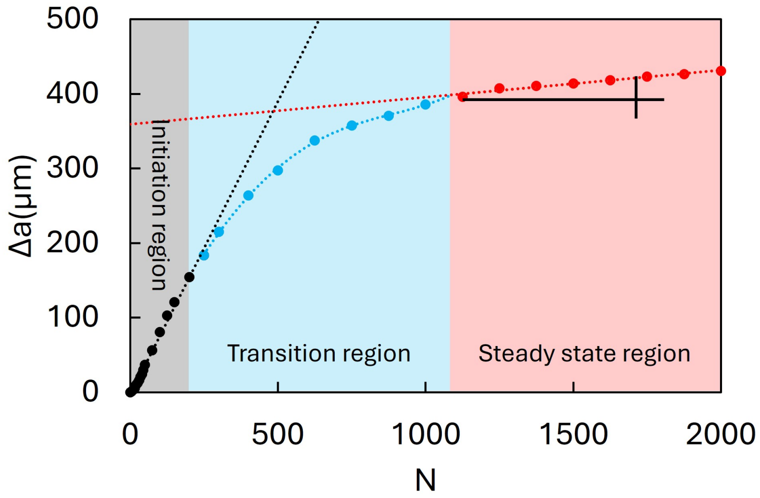

Figure 1: Crack growth curve for hydrogel at $G = 3.57$ J/m^2, showing initiation, transition, and steady-state regimes. The red dashed line represents the linear interpolation of the data in the steady-state regime, used to calculate the crack propagation rate under cyclic loading for the given conditions.

Supplementary Figure S2 – Evolution of the energy release rate during cyclic loading

To determine how the driving force for fracture changes during testing, the evolution of the applied energy release rate G, calculated using the CTOD method, was evaluated for hydrogel and PDMS, as presented in Figure 2a and 2b, respectively. In both materials, a $\sim$10% decrease in G is observed in experiments without significant crack advance (yellow curves for hydrogel and purple curves for PDMS). This reduction may arise from crack closure effects or local crack-tip softening induced by the compressive zone.

When crack propagation occurs close to the threshold energy, the measured G remains nearly constant in PDMS (yellow and green curves) and shows only a minor decrease during the initial cycles in hydrogel (black curve). However, for cases with relatively large crack extension, the measured energy release rate decreases more noticeably (red and blue curves for hydrogel and red curve for PDMS). We attribute this to the influence of tensile plastic deformation near the crack tip and compressive plastic wak behind the crack tip, which modifies the CTOD geometry and reduces the effective energy release rate.

If the extent of plastic deformation is small, as in PDMS, the effect on CTOD is negligible. In hydrogel, plastic deformation has a limited but measurable impact. This observation supports our interpretation that the nonlinear regime occurs within a very narrow window at the beginning of the experiment, during which crack propagation is extremely small and may be missed. For hydrogel, the onset of this nonlinearity in G-evolution is typically detected after 100–150 cycles. In the subsequent figures, the relation between G evolution and the development of plastic zones will be further analyzed.

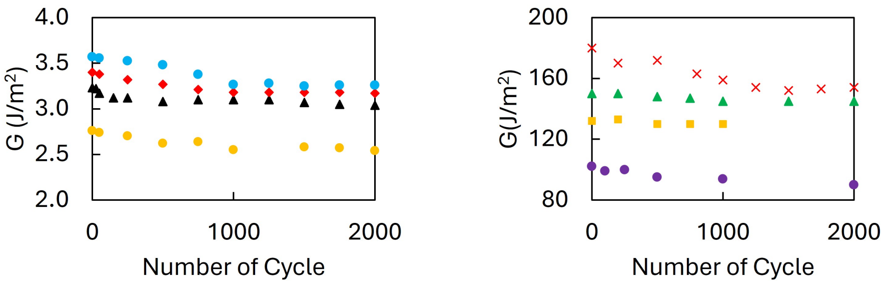

Figure 2: Evolution of the energy release rate G during cyclic loading, calculated from CTOD, for (a) hydrogel and (b) PDMS. (a) Hydrogel experiments: yellow curve shows no crack propagation, black curve corresponds to very limited crack growth near threshold, and red/blue curves correspond to larger crack extensions. (b) PDMS experiments: purple curve shows no crack propagation, yellow and green curves correspond to near-threshold cases with minimal growth, and the red curve represents a case with large crack propagation. For both panels, colors and symbols match those used in Figure 2 of the main text.

Supplementary Figure S3 – Strain field evolution in hydrogel without crack propagation

To investigate local deformation mechanisms below the fatigue threshold, the y-direction strain field near the crack tip in hydrogel was mapped under sub-threshold loading at two different applied energy release

rates, as shown in Figure 3. Panels (a–c) correspond to $G = 2.76$ J/m^2, while panels (d–f) correspond to $G = 3.0$ J/m^2, each shown at the 100th, 1000th, and 2000th loading cycles, respectively.

In both cases, no visible crack propagation is observed; however, a compressive strain zone develops ahead of the crack tip and grows with the number of cycles. At $G = 2.76$ J/m^2, the compressive zone is limited to a small region immediately in front of the crack tip. At $G = 3.0$ J/m^2, the compressive zone extends over a noticeably larger area, confirming that both the magnitude and spatial extent of this zone depend on the applied energy release rate.

These results strengthen our main-text conclusion that plastic deformation accumulates even in the absence of crack propagation. Importantly, the observation of compressive zones under two different energy release conditions demonstrates that this phenomenon is robust and reproducible, rather than a random or isolated event. The dependence of compressive zone size on G directly supports the statement in the main text that "the compressive zone value and size depend on the energy release rate without crack propagation."

This behavior highlights a key difference between soft brittle polymers and classical elastic models: even below the fatigue threshold, hydrogels undergo irreversible local deformation. Such compressive zones resemble crack closure and shielding mechanisms well-documented in metals , suggesting a soft-material analog of these fatigue processes.

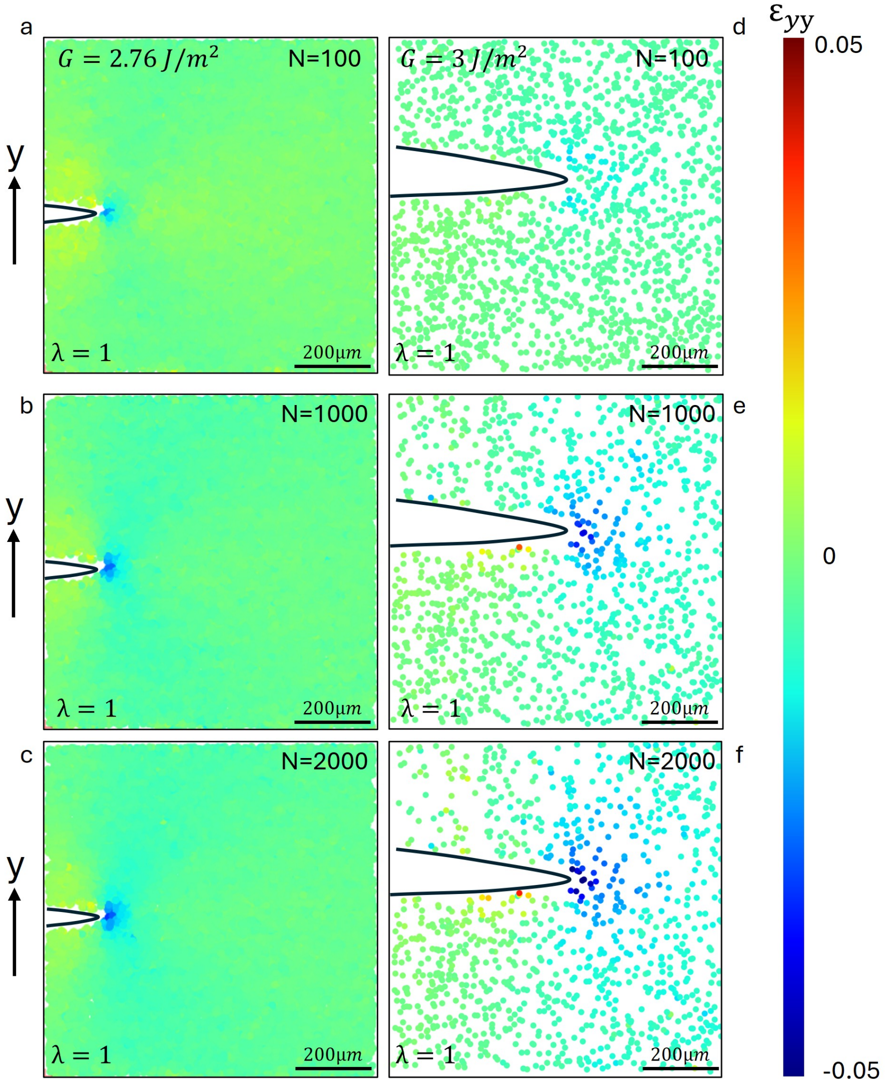

Figure 3: Strain field evolution in hydrogel without crack propagation. (a–c) $G = 2.76$ J/m^2 at the 100th, 1000th, and 2000th cycles. (d–f) $G = 3.0$ J/m^2 at the 100th, 1000th, and 2000th cycles. In both cases, a compressive zone develops ahead of the crack tip and grows with cycling. The zone is more extended and intense at higher G, confirming that both the size and magnitude of the compressive field increase with cycle number and applied energy.

Supplementary Figure S4 – Swelling analysis of hydrogel

To test whether the compressive zones observed near the crack tip could be explained by swelling or deswelling in the water bath, the strain components ε_{xx}, ε_{yy}, ε_{zz}, and the volumetric Jacobian J for hydrogel were evaluated under sub-threshold cyclic loading, as shown in Figure 4. If swelling were the dominant effect, J should follow the same trend as ε_{yy}. Instead, J remains small and more closely resembles ε_{zz}. This confirms that volumetric changes due to swelling or deswelling are negligible. Therefore, the compressive zones in hydrogel must originate from polymer-level damage mechanisms rather than swelling effects.

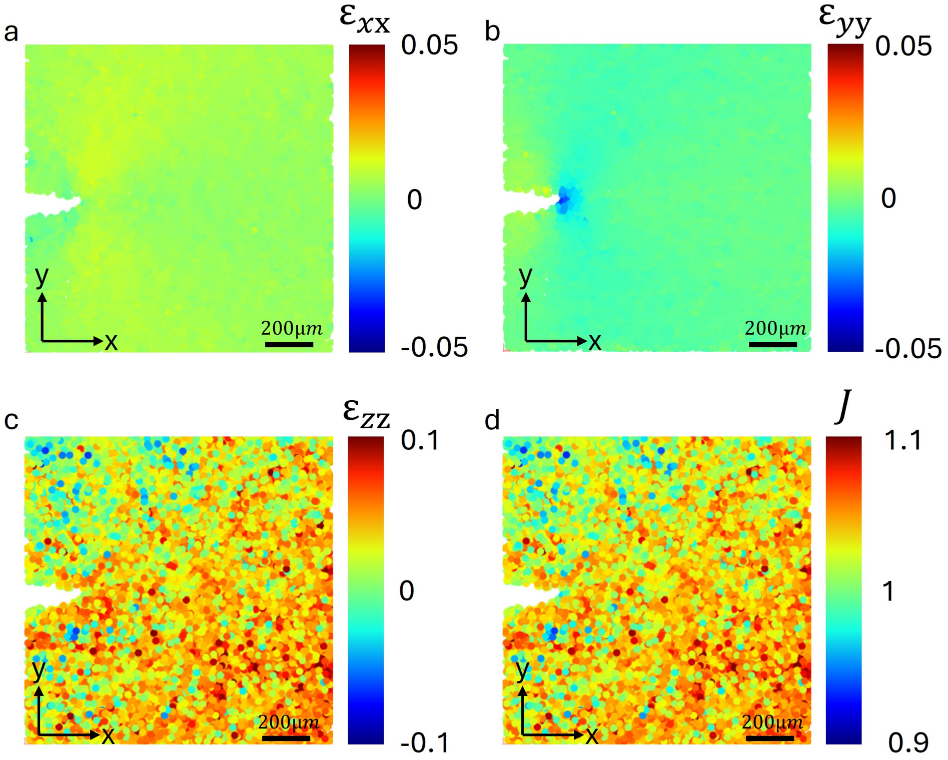

Figure 4: Swelling analysis of hydrogel at $G = 2.76$ J/m^2. (a) ε_{xx}, (b) ε_{yy}, (c) ε_{zz}, and (d) volumetric strain J. The small values of J and its correlation with ε_{zz} confirm that swelling/deswelling is not responsible for the compressive zones. This supports the conclusion in the main text (Figs. 3–4) that the compressive fields arise from polymer-level damage.

Supplementary Figure S5 – Swelling analysis of PDMS

As with hydrogel, an identical analysis was performed to evaluate whether swelling or deswelling effects contribute to the observed compressive zones in PDMS. The strain components ε_{xx}, ε_{yy}, ε_{zz}, and the volumetric Jacobian J for PDMS under sub-threshold cyclic loading were quantified, as shown in Figure 5. The

results show that J remains small and does not follow the behavior of ε_{yy}. Instead, J correlates more closely with ε_{zz}, indicating negligible volumetric changes. Consequently, swelling or deswelling cannot explain the compressive zones in PDMS; their origin must also lie in polymer-level damage processes.

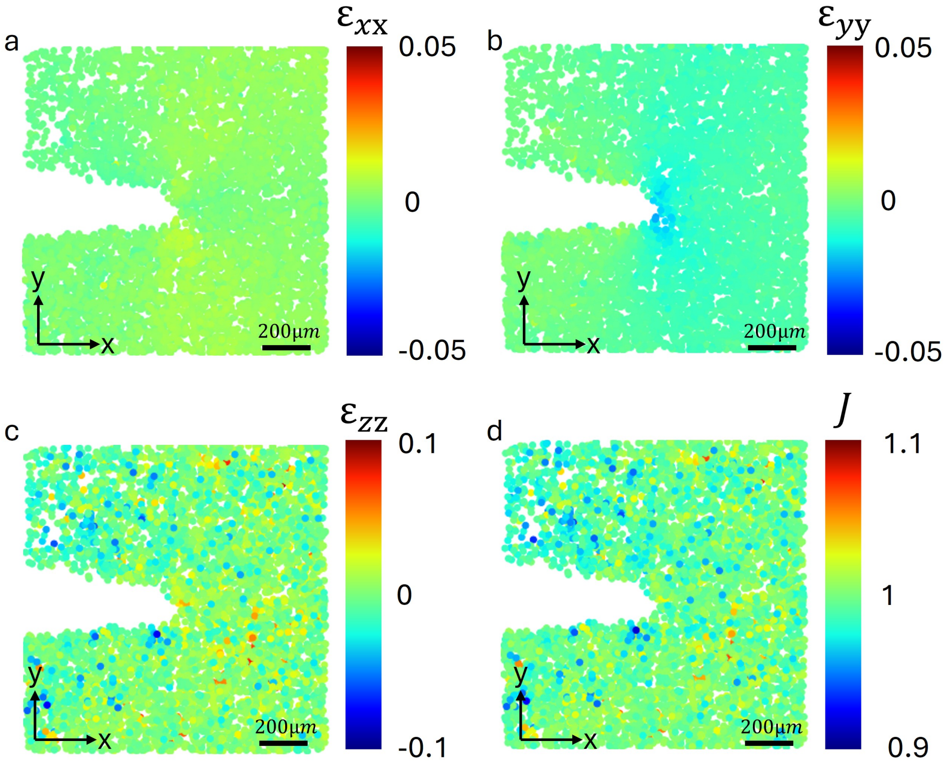

Figure 5: Swelling analysis of PDMS at $G = 102$ J/m^2. (a) ε_{xx}, (b) ε_{yy}, (c) ε_{zz}, and (d) volumetric strain J. The absence of swelling/deswelling effects indicates that compressive zones result from polymer-level damage mechanisms, consistent with the interpretation in the main text (Figs. 3–4).

Supplementary Figure S6 – Strain field evolution in hydrogel with crack propagation

To contrast with the sub-threshold behavior, the mechanisms of damage accumulation accompanying actual crack growth were also examined. The y-direction strain field near the crack tip in hydrogel was visualized during cyclic loading at two different applied energy release rates, as shown in Figure 6. Panels (a–c) correspond to $G = 3.23$ J/m^2, while panels (d–f) correspond to $G = 3.4$ J/m^2, each displayed at selected cycle numbers. In both cases, the crack propagates under cyclic loading, and a tensile plastic zone develops ahead of the crack tip. At $G = 3.23$ J/m^2, the tensile plastic zone is relatively small, and the crack tip opening displacement (CTOD) decreases only slightly with cycling. By contrast, at $G = 3.4$ J/m^2, the

tensile plastic zone expands significantly, leading to a measurable reduction in CTOD with increasing cycle number.

These results demonstrate that the size of the tensile plastic zone directly affects the apparent CTOD measurement. A larger plastic zone modifies the local crack-tip geometry, thereby reducing the effective CTOD and the measured energy release rate. In addition, a compressive wake forms behind the crack tip, further altering crack-tip geometry and contributing to the observed decrease in G. This finding supports the interpretation in the main text that plastic deformation influences G-evaluation once its extent becomes significant.

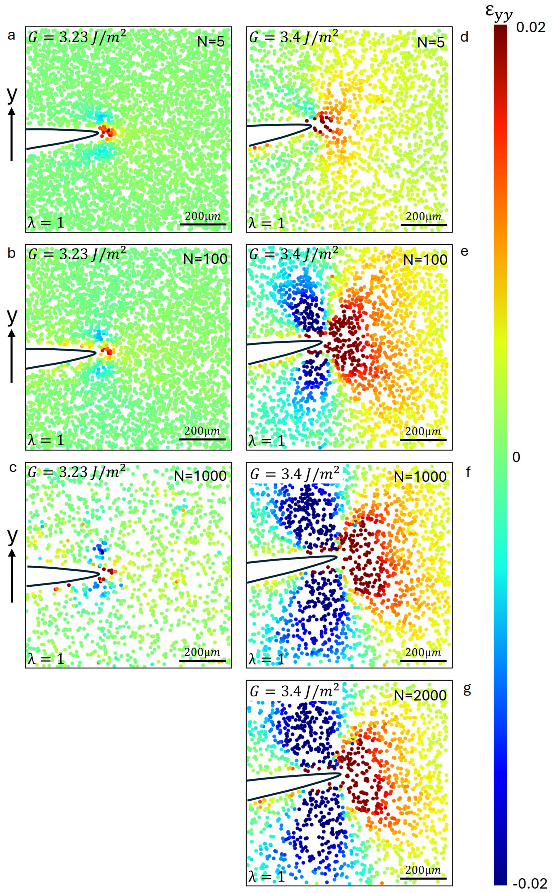

Figure 6: Strain field evolution in hydrogel during cyclic crack propagation. (a–c) $G = 3.23$ J/m^2: a small tensile plastic zone develops ahead of the crack tip, with minor growth in magnitude but little change in size; CTOD decreases only slightly. (d–f) $G = 3.4$ J/m^2: a much larger tensile plastic zone forms and grows significantly in both size and magnitude with cycling. A compressive plastic wake is also observed behind the crack tip, which alters the crack-tip geometry and contributes to the reduction of CTOD.

Supplementary Figure S7 – Strain field evolution in PDMS with crack propagation

A similar investigation into crack growth conditions was conducted for the second material. The y-direction strain field near the crack tip in PDMS was mapped during cyclic loading at three different applied energy release rates: 132, 150, and 180 J/m^2, as shown in Figure 7. Panels (a–c), (d–g), and (h–k) correspond to these three cases, respectively. At $G = 132$ and 150 J/m^2, crack propagation occurs with only a small tensile plastic zone ahead of the crack tip. In these cases, the tensile zone remains limited in size, so the crack tip opening displacement (CTOD) is essentially unaffected and the measured energy release rate G remains constant. By contrast, at $G = 180$ J/m^2, the tensile plastic zone expands significantly in both size and magnitude. This expansion modifies the local crack-tip geometry and leads to a measurable decrease in CTOD, resulting in a reduction of the effective energy release rate. A larger compressive plastic wake is also observed behind the crack tip at this higher energy, further contributing to the CTOD reduction. These findings confirm that, as in hydrogel, the influence of plastic deformation on CTOD in PDMS depends on the size of the tensile plastic zone. Only when the zone becomes sufficiently large does it affect G-evaluation; otherwise G remains stable.

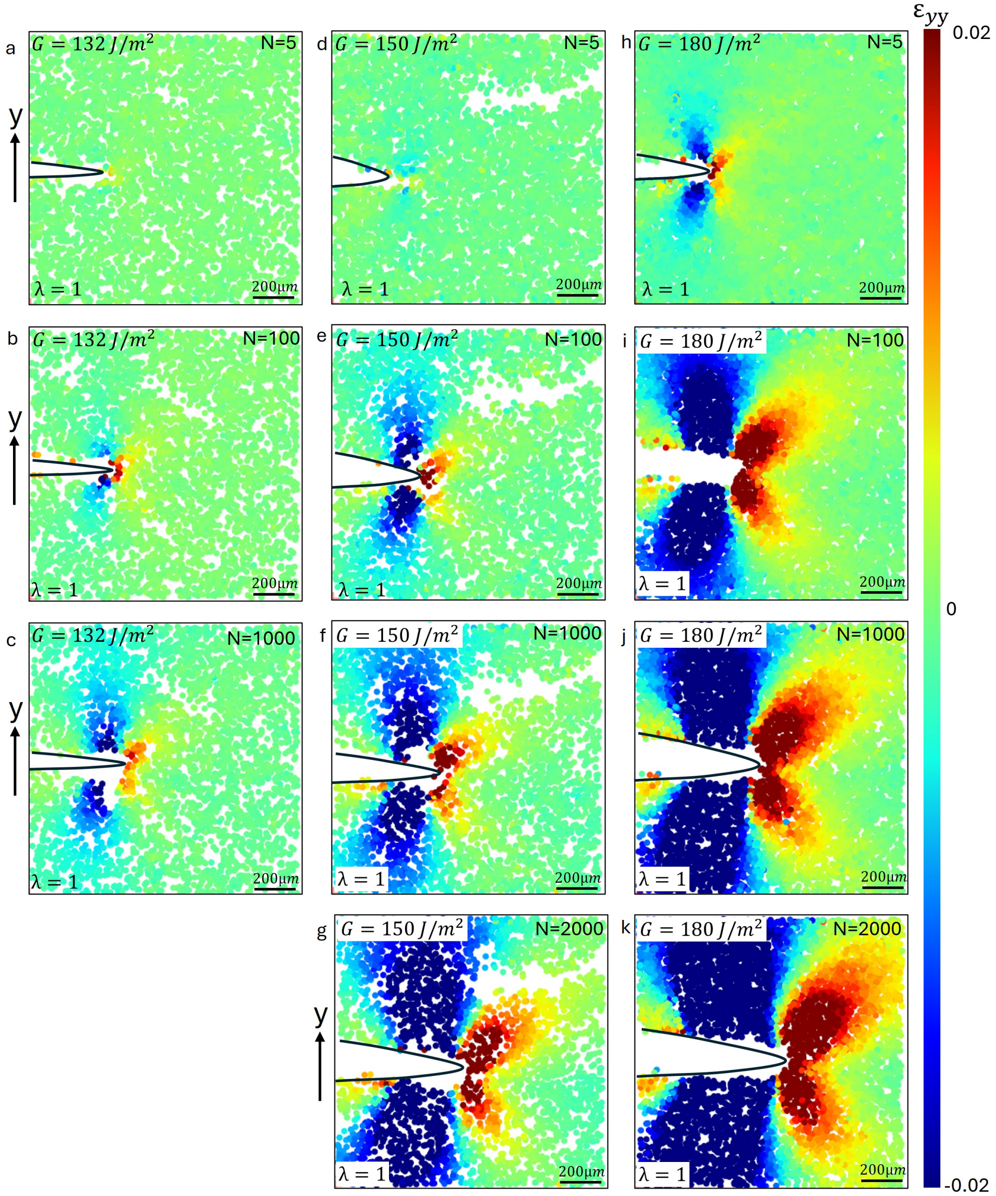

Figure 7: Strain field evolution in PDMS during cyclic crack propagation at three applied energy release rates. (a–c) $G = 132$ J/m^2: a small tensile plastic zone forms, with negligible influence on CTOD, so G remains constant. (d–g) $G = 150$ J/m^2: the tensile zone remains limited, and G is again stable. (h–k) $G = 180$ J/m^2: a large tensile plastic zone develops ahead of the crack, accompanied by a compressive wake behind it, leading to a reduction in CTOD and the effective energy release rate.
